# Red meat—an essential partner to reduce global greenhouse gas emissions

**DOI:** 10.1093/af/vfaa035

**Published:** 2020-10-30

**Authors:** Thomas M Davison, John L Black, Jonathan F Moss

**Affiliations:** 1 Livestock Productivity Partnership, University of New England, Armidale, New South Wales, Australia; 2 John L. Black Consulting, Warrimoo, New South Wales, Australia; 3 UNE Business School, University of New England, Armidale, New South Wales, Australia

**Keywords:** carbon neutral, methane, ruminants

**Implications**

- Recent work in Australia has shown the possibility of a carbon neutral red meat industry in a program titled Carbon Neutral 2030. This paper summarizes options to significantly reduce methane emissions from ruminants given this is the major greenhouse gas from Australian agriculture.
- Research has identified the key role of novel supplements, anti-methanogenic legumes and rumen microbial manipulation.
- Novel ruminant supplements such as marine macro algae (*Asparogopsis* spp.) at low dietary levels could significantly mitigate methane emissions in farming systems with improved productivity and no detectable impacts reported for animal health or meat quality.
- This article demonstrates how the energetic pathways for methane abatement within the rumen can be manipulated, with potential intergenerational impact on herd methane emissions as a potential future strategy.
- Economic analysis investigates productivity and carbon abatement potential for a range of methane mitigation strategies.

## Introduction

Management of ruminant livestock for red meat consumption is a major human enterprise. Approximately 1.3 billion people depend partially, or entirely, on livestock for their livelihoods. Given population projections and rising living standards in developing nations, the Food and Agriculture Organization (FAO, 2017) projects that demand for red meat from ruminants will continue to increase at the rate of around 1.5% per year. However, at a time of concern about the negative impacts of global warming, this reliance on red meat and the associated methane emissions has caused considerable debate on its role for humanity (Bryngelsson et al., 2016).

If increasing the supply of red meat is to be part of the solution for increasing food production for the growing population, solutions must be found to reduce methane emissions and produce less greenhouse gas (GHG). According to the FAO, ruminant supply chains produces 5.7 billion tons of carbon dioxide equivalents per year, which represents 80% of all livestock emissions globally and 16% of total world emissions. Cattle make up 80% of ruminant emissions (Gerber et al., 2013). Methane is a potent greenhouse gas, which is 28 times more powerful than carbon dioxide in global warming potential (IPCC, 2014).

This article uses Australian and international research to describe options to substantially mitigate methane emissions from ruminants and outlines ways for the Australian industry to become carbon neutral. Research in Australia over the last 15 yr has investigated the biology of enteric methane production and examined a range of potential methods for managing methane emissions. Lowering GHG emissions or sequestering carbon in grazing systems and feedlot enterprises have been separately explored with the aim of making the Australian red meat industry carbon neutral by 2030 (Mayberry et al., 2019). Although grazing production systems predominate in Australia, the research outlined in this paper indicates options for other red meat industries at a global scale.

## Greenhouse Gas Emissions in Australia

**Figure 1.**
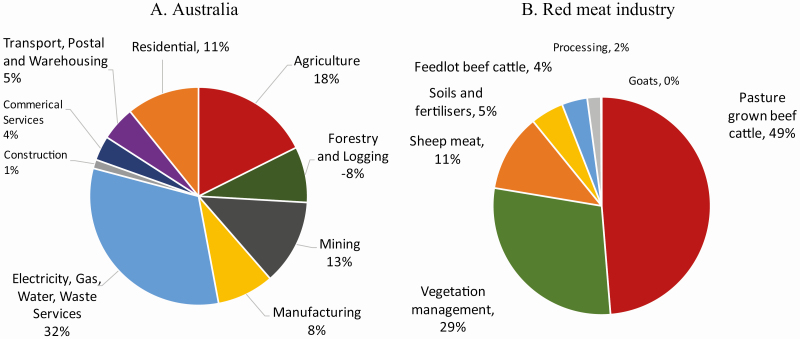
National GHG Inventory emissions—Australia and the red meat industry in 2017 (Derived from Department of Industry, Science, Energy and Resources, Australian Greenhouse Emissions Information System).

Agriculture represented 18% of Australian GHG emissions during 2017 (Figure 1A), with approximately 15% coming from the red meat industry. The red meat sector of beef, sheep, and goats is important economically to Australia, where it generates around US$21 billion in off-farm value and contributes approximately US$17 billion to Australian exports. Around 64% of GHG from the red meat industry is from methane emissions from ruminants through microbial fermentation of feed, with vegetation management and land clearing accounting for another 30% (Figure 1B). The majority (78%) of the methane emissions are from pasture raised beef, followed by 18% from sheep, 4% from feedlots, and <1% from goats. Ruminant methane enteric emissions comprise approximately 10% of accountable GHG emissions in Australia.

## Methane Production Within the Rumen

Methane is produced in the rumen of cattle under anaerobic conditions by a specialized group of organisms called *Archaea*, which are a separate phylogenic kingdom from bacteria and eukaryotes (Woese et al., 1990). Methanogenic *Archaea* scavenge products within the rumen produced by the populations of bacteria, protozoa, and fungi through the fermentation of feed. Methane is produced by several groups of methanogens, which use different substrates within the rumen. Hydrogenotrophic methanogens produce methane from hydrogen and carbon dioxide, and methylotrophic methanogens produce methane from free methyl groups that come from plants and other microbial sources, whereas small amounts of methane are produced by acetoclastic methanogens from acetic acid. The number of protozoa in the rumen also contributes to methane production because they produce substantial amounts of hydrogen when digesting feed (Nguyen et al., 2020). The amount of methane produced from the fermentation of feed depends on the concentration of substrates available and ranges from 2% to 12% of digested energy (Johnson and Johnson, 1995). On average, each cow emits 55 to 67 kg of methane or 1,375 to 1,675 kg CO_2_ equivalents a year. This represents a loss of around 35 d a year of digested energy, assuming a cow eats 10 kg/d of a forage with 65% digestibility and 12% of digested energy is lost as methane. Capture of this energy through methane mitigation strategies would enhance ruminant productivity.

Animals consuming concentrate diets have a lower methane output per unit of energy digested than animals consuming high-fiber diets. The proportion of digested energy lost as methane is determined by the specific biochemical pathways occurring within the rumen and these pathways are now well understood. Changing the proportion of fermented energy that passes through each pathway will substantially alter the amount of methane produced and the efficiency of energy use by the animal.

**Figure 2.**
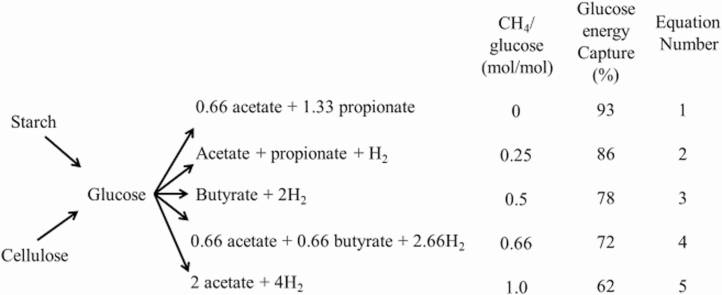
The five dominant pathways for glucose fermentation in the rumen. CH4 is methane, H_2_ is hydrogen. Adapted from Janssen (2010).

The primary carbohydrates fermented in the rumen by microorganisms are starch, cellulose, and hemicelluloses. Both starch and cellulose consist of chains of glucose molecules linked either by 1–4 α-bonds in the case of starch or 1–4 β-bonds for cellulose. Glucose is a primary substrate for microorganisms within the rumen. Glucose can be degraded by five competing pathways to produce volatile fatty acids, primarily propionate, acetate, and butyrate, which are the main energy substrates for ruminants. These pathways contribute to the production of different amounts of methane and have different efficiencies of energy conversion from glucose to volatile fatty acids (Figure 2). The reactions are numbered 1 to 5 from the highest to lowest energy efficiency. The bottom pathway (5) produces the most methane and has an efficiency of conversion of glucose energy to volatile fatty acid energy of 62%, compared with the top pathway (1), which produces no methane and has an efficiency of conversion of glucose energy to volatile fatty acids of 93%. Each species of microorganisms within the rumen has a predominant pathway, but trace amounts of other products can be formed using the alternate pathways. The dominant pathway for a microorganism depends on the substrate metabolized and the redox state of the rumen, which is negatively correlated with hydrogen concentration, acidity or pH (Huang et al., 2018).

**Figure 3.**
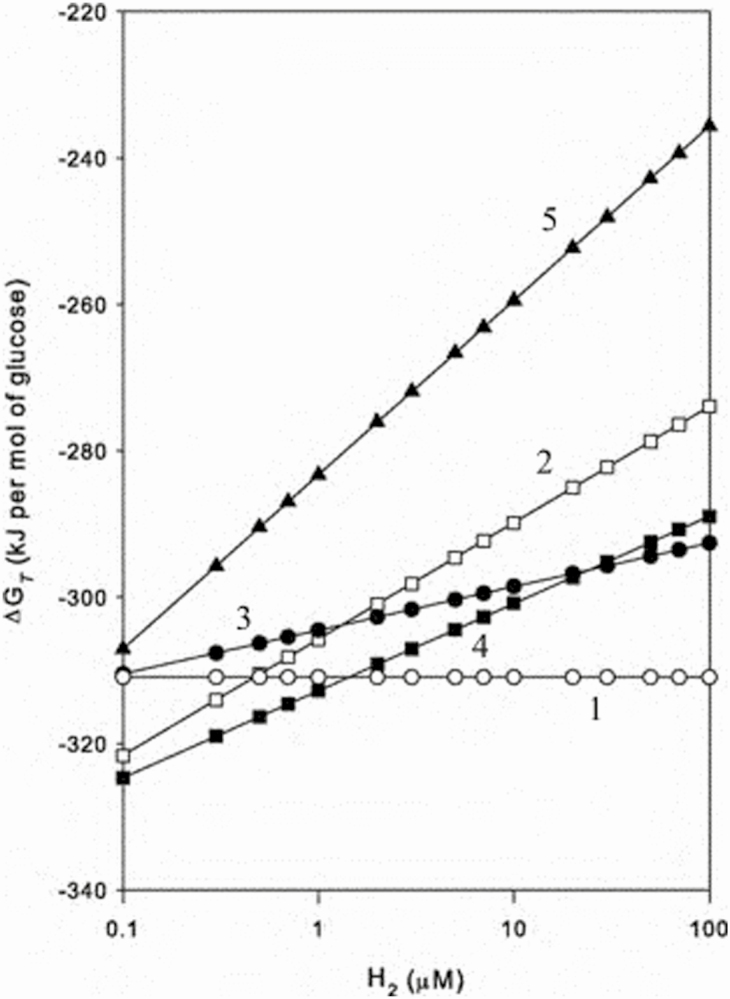
The effect of hydrogen concentration in the rumen on the Gibbs free energy change of competing biochemical pathways for glucose metabolism in the rumen. Reactions with low Gibbs free energy dominate. Adapted from Janssen (2010).

A major factor driving the competition between these biochemical pathways is known as Gibbs energy dissipation, or free energy change, with lower free energy pathways being preferred. Janssen (2010) shows that the Gibbs energy dissipation of the five reactions change with hydrogen concentration in the rumen (Figure 3). The free energy change of reaction 1, which produces no methane, is unaffected by hydrogen concentration. However, hydrogen concentration markedly alters the free energy change of pathway 5, which produces the highest methane and has the lowest energetic efficiency. The impact of hydrogen concentration on the free energy change of other pathways is intermediate between pathways 1 and 5. The high methane producing and low energetic efficiency pathways 4 and 5 will dominate when hydrogen concentrations in the rumen are low.

Hydrogen concentration in the rumen can be as low as 0.001 µM when animals are fed diets high in fiber. This low hydrogen concentration results in a predominance of metabolism through pathways 4 and 5 and explains why methane output is high from digested energy when cattle are consuming high-fiber diets. The hydrogen concentration in the rumen increases when animals are fed high-grain diets, typical of intensive finishing systems. These elevated rumen hydrogen concentrations shift the metabolic pathways towards a predominance of pathway 1, with low methane production as is observed when cattle are fed concentrate diets. Predominance of the different microbial species and biochemical pathways is one reason why it is more energetically efficient to finish cattle with high-grain diets rather than pasture. Supplements, which have been shown to reduce methane emissions, such as the red marine macroalgae (seaweed), *Asparagopsis* spp., chloroform, bromochloromethane, and 3-nitrooxypropanol (3-NOP) also result in higher hydrogen concentrations in the rumen. Research into the mechanism of action of 3-NOP may suggest that the increase in hydrogen is due to 3-NOP reacting with reduced vitamin B12, which inhibits the cobamide-dependent enzyme methyl-coenzyme (CoM) reductase step in methanogenesis and blocking the synthesis of methane (Duin et al., 2016).

An increase in hydrogen production in the rumen through, for example, the metabolic activity of protozoa, will increase methane synthesis by hydrogenotrophic archaea. The concentration of hydrogen in the rumen becomes a balance between the amount produced and the amount incorporated into methane and other products. When methane emissions are reduced, at least in continuous culture systems, a portion of the increased rumen hydrogen is incorporated into formate as precursor for propionate and into greater microbial growth. However, a recent culture experiment by Ungerfeld et al. (2019) did not consistently show enhanced microbial growth with methane inhibition.

## Mechanisms to Reduce Methane Emissions

**Figure 4.**
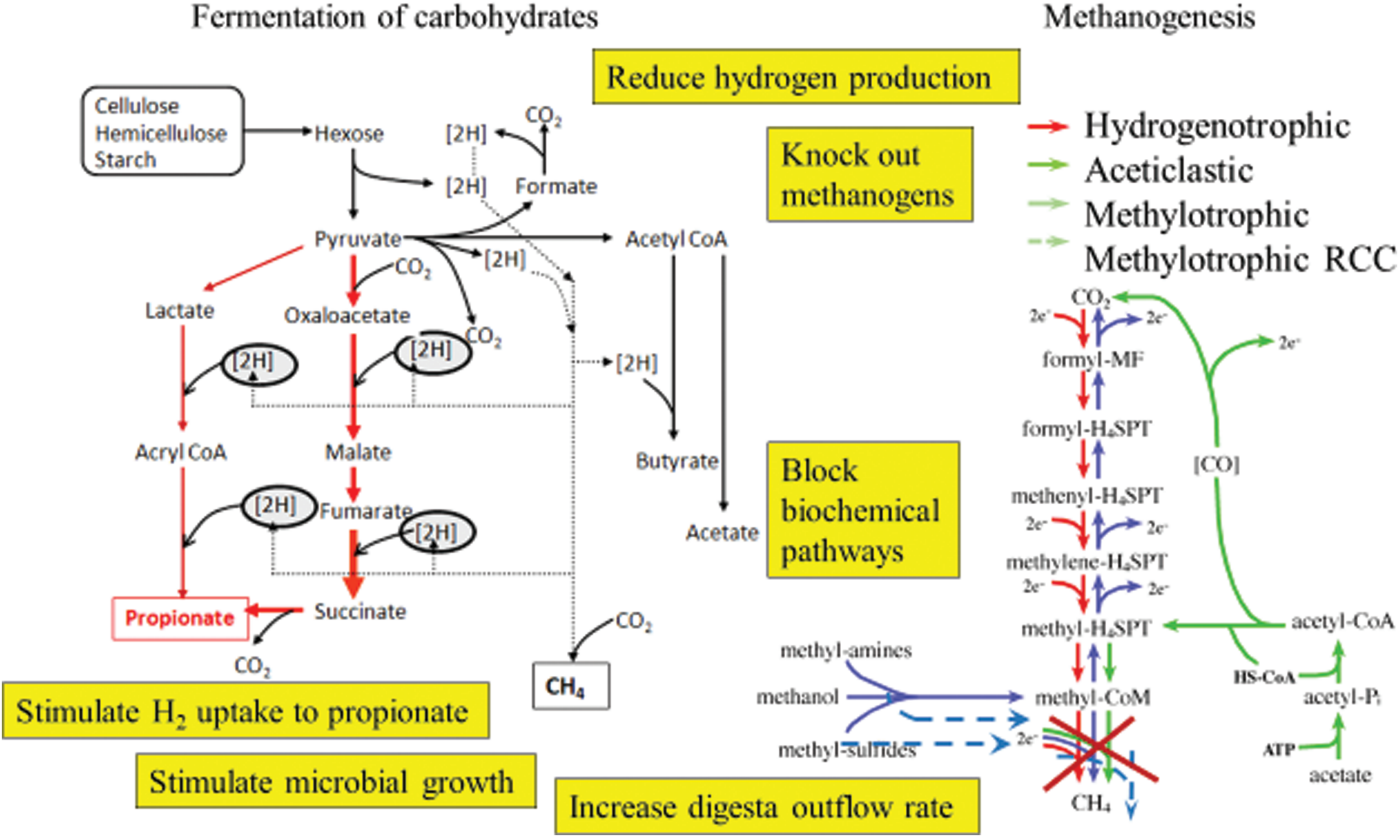
Potential approaches to reducing methane emissions from ruminants. Illustration of the biochemical pathways for methanogenesis was from Galagan et al, (2002).

The predominant methods for managing methane emissions (Figure 4) are to:

- decrease total hydrogen production within the rumen by practices including removal of protozoa, feeding high-grain diets, and decreasing proportion of feed degraded in the rumen;
- increase hydrogen concentrations within the rumen and stimulate hydrogen uptake by bacteria through enhancing more energetically efficient biochemical pathways that produce propionate;
- remove the methanogens by vaccination or other processes;
- interrupt biochemical pathways for methanogenesis such as the cobamide-dependent enzyme methyl-coenzyme reductase step as occurs with the 3-NOP compound;
- increase the rate of rumen emptying as occurs in sheep selected genetically for low methane output (Goopy, 2019) or increasing rumen osmolality;
- enhance the conversion of fermented substrates into microbial growth rather than volatile fatty acid production by ensuring sufficient dietary nitrogen and sulfur.

## Summary of Methane Mitigation Technologies

The National Livestock Methane Program (NLMP) in Australia operated from 2009 to 2016 and investigated the effectiveness of a range of strategies to reduce methane emissions from ruminants (MLA 2016).

The NLMP investigated:

- genetic selection of beef cattle and sheep for low methane emissions;
- feed supplements including grape marc, nitrate, bioactive compounds from Australian leptospermum and melaleuca species, marine and freshwater macro algae and 9 kg/d of wheat to dairy cows;
- feeding forages including the grazing of *Leucaena* spp. by cattle, high productivity and anti-methanogenic temperate legumes, *Biserrula pelecinus,* and Australian native shrubs, *Eremophila glabra* and *Atriplex nummularia*, with known anti-methanogenic properties to sheep;
- understanding rumen function and its manipulation to reduce methane emissions.
- In addition to the research conducted, published information on other methane mitigation strategies were evaluated including:
- vaccination against methanogens;
- feeding 3-NOP and biochar supplements;
- best management practices for grazing temperate pastures for maximum pasture utilization.

Research findings since 2016 have been included in this paper. An evaluation of each mitigation strategy was undertaken to estimate:

- mitigation potential for individual animals and across Australia;
- impact on animal productivity;
- likely cost and barriers to implementation;
- time to implementation on production enterprises.

**Figure 5.**
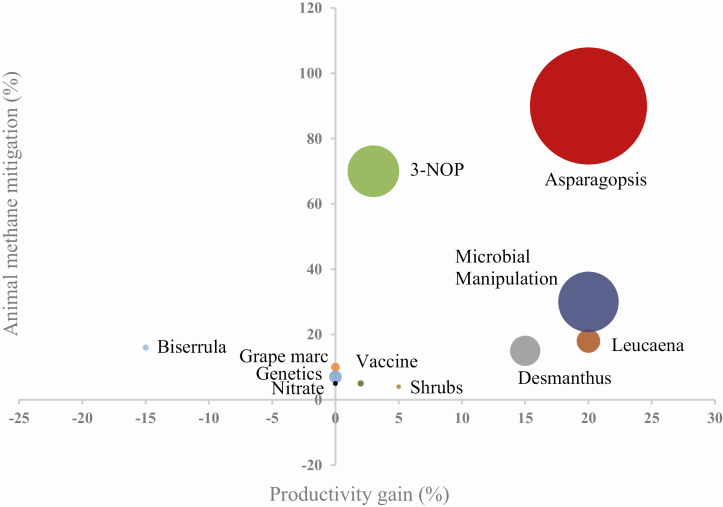
Relationship between the methane mitigation potential in individual animals and estimated productivity gain for a range of methane mitigation strategies examined. The size of the bubble-dot represents the proportion of total Australian GHG reduction attributable to each mitigation strategy. The following assumptions based on a consensus of livestock producers, scientists, and science administrators in 2015 were used for 1) the proportion of the national ruminant population for which a strategy is applicable and 2) the likely adoption rate, respectively: Genetics, 100%, 3%; Grape marc, 2%, 50%; Asparagopsis, 100%, 20%; Nitrate, 4%, 5%; 3-NOP, 100%, 5%; Leucaena, 20%, 20%; Desmanthus, 40%, 20%; Shrubs, 5%, 5%; Microbial manipulation, 80%, 20%; Vaccination, 100%, 1%; Biserrula, 3%, 10%. The numbers would be expected to change depending on the price for CO_2_ equivalents.

The estimated likely methane reduction potential for an individual animal, the potential change in productivity, and impact on the contribution to a carbon neutral industry is shown as a bubble diagram for each mitigation strategy in Figure 5.

The red seaweed, *Asparagopsis* spp., retains in specialized cellular structures around 20 bromoform compounds, which can be released for self-protection. Similar bromoform compounds have been known to reduce methane emissions. Recent research into the effects of *Asparagopsis* spp. supplementation on methane mitigation indicate up to a 98% reduction in methane emissions from Brahman-Angus cattle fed 0.2% of organic matter as *Asparagopsis* spp. in a high-grain diet (Kinley et al., 2020). No detectible levels of bromform or dibromochloromethane have been identified in muscle or fat nor have there been adverse effects on animal health (Li et al., 2018; Kinley et al., 2020). Additional research is being undertaken to select *Asparagopsis* spp. strains with higher anti-methanogenic properties and to develop commercial methods for growing viable quantities. Studies with 3-NOP suggest that methane emission reductions of up to 70% is possible (McGinn et al., 2019), but the compound needs to be provided continually and is best fed as part of a mixed ration. DSM Nutritional Products Ltd., the owner of 3-NOP, is currently seeking registration for use in ruminant diets in various jurisdictions around the world.

Research to date on legumes for grazing systems such as *Leucaena* spp. and *Desmanthus* spp. has shown potential methane abatement of 10% to 20% where they account for 10% to 50% of the diet. This is similar to the results previously found for *Biserrula* spp., but contrary to the tropical legumes, growth rate of sheep was reduced by up to 20% compared with traditional pastures (Vercoe, 2016). Legumes work within specific climate and soil regions, so proof of concept will be required across regions in any country where their anti-methanogenic potential may be applicable. The advantage of legumes is their high impact on productivity and potential carbon sequestration (Fornara and Tilman, 2008; Radrazinni et al., 2011); however, compared with *Asparagopsis* spp. and 3-NOP, legumes, or other forages found to be anti-methanogenic will be less efficient at reducing methane emissions.

Manipulation of rumen microbial populations appears to have long-term, intergenerational possibilities for substantially reducing methane emissions and increasing the efficiency of energy utilization. The experiment of Abecia et al. (2013) is a classic example of manipulating rumen microbial populations in ruminants. There were two groups of female goats, with each doe having twins. One group of does and one twin kid from each doe was fed the methane mitigation agent, bromochlormethane, for 2 mo from birth to weaning. Methane emissions from the kids were compared with untreated controls for up to 4 mo after cessation of the treatment. Animals in each treatment were kept isolated from one another to prevent cross contamination of rumen microbial populations. One month after cessation of the treatment, kids from both treated and untreated does produced on average 55% less methane than the untreated kids. However, 4 mo after cessation of the treatments only kids from treated does produced 33% less methane and grew 20% faster than the other treatments. Cross contamination of rumen microbes from the untreated does was presumably responsible for their treated kids reverting to high methane producers. This experiment showed that changes in microbial populations favoring reduced methane emissions and increased propionate synthesis can be maintained for long periods, but only when cross contamination of rumen microbial populations from untreated animals is avoided. Similar long-term effects on methane emissions and microbial populations have been observed in calves treated with 3-NOP for up to 3 wk post-weaning (Meale et al., 2019). The possibility generated from these experiments is for whole herds of animals with desired rumen populations to be created and maintained through generations provided they are isolated from animals with different rumen populations. Marked changes in rumen microbial populations in the desired direction for increasing propionate and reducing methane production also occur following feeding of high-grain diets, which increase rumen hydrogen concentrations (Janssen, 2010). Similarly, the induced changes in microbial populations would be expected to be maintained, provided the animals were located with others similarly treated.

Breeding ruminants for low methane could be considered a poor investment because of the large amount of research still required and the relatively small mitigation potential of around 7% above selection productivity gains obtained through existing genetic improvement schemes (Fennessy et al., 2018). Vaccination against methanogens would appear to be a simple management tool for reducing methane emissions by around 8% for all ruminants (Wright et al., 2004); however, no published results could be found to reinforce this view. Similarly, changing wheat supplements from 6 to 9 kg for dairy cows reduced methane by 30% to 40% and also reduced milk fat but has not been widely adopted (Moate et al., 2020), because the methane mitigation effects disappear over time. Extraction of bioactive compounds from Australian leptospermum and melaleuca species remains prospective, but no research has been done since 2016 to determine viability of these compounds for methane mitigation. Biochar, produced from organic material burned under low oxygen, indicated potential from an earlier animal study (Leng et al., 2012) and laboratory studies (Schmidt et al., 2019); however, recent research has not demonstrated reductions in methane emissions when fed to feedlot cattle (Terry et al., 2019). There are likely to be practical and synergistic benefits from combining strategies for reducing enteric methane emissions as proposed by Beauchemin et al. (2020), because they have different biological activities.

## Methane Mitigation Technologies With Greatest Potential

For ruminant systems, the greatest practical potential for reducing methane at this stage of our knowledge is concluded to be:

- supplementation of anti-methanogenic compounds and bioactives—*Asparagopsis* spp. and 3-NOP;
- forage legumes—*Leucaena* spp. and *Desmanthus* spp.;
- rumen microbial manipulation—potential to manipulate the rumen microbial populations for long-term and intergenerational methane mitigation and improved productivity.

## Economic Value of Most Promising Methane Mitigation Technologies

**Figure 6.**
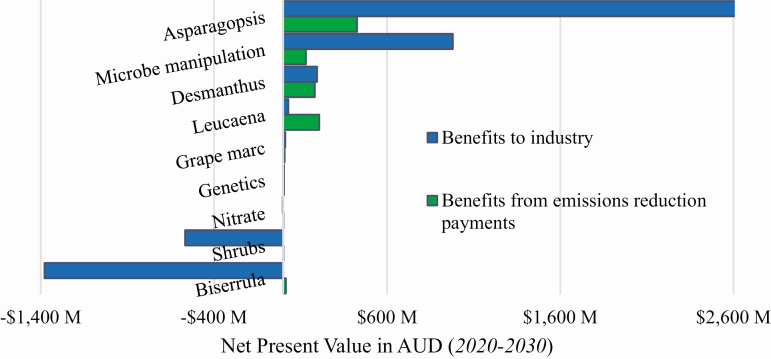
Economic value of the potential technologies to reduce emissions from the red meat sector in Australia.

An economic evaluation of the benefit of each of these technologies was undertaken to identify which have the greatest financial prospect for the Australian red meat industry up to 2030 (Figure 6). The net present value for each of the technologies was calculated as the discounted additional benefits associated with a technology minus the discounted additional costs multiplied by the assumed adoption rates. Benefits of these technologies may include financial gains from improvements to productivity and also from the sale of carbon credit units for verifiable activities that reduce emissions below baseline levels. Figure 6 provides a depiction of the overall benefits to the industry (blue bars) and the value of the benefits that are attributable to the sale of carbon credit units (green bars). Net present value for 3-NOP was not calculated as this product is a commercial product that has not yet been released and no estimates of the possible costs were available.

The technologies with the highest net present value include the use of supplements such as *Asparagopsis* spp. and the increased use of forage legumes. The use of grape marc, genetics, and nitrates only has the potential for minor financial benefit to the industry. The planting of Australian native shrubs for forage and the forage legume *Biserrula* have costs that far outweigh the benefits received. This confirms the areas with the highest potential benefit from further research and development include the use of supplements, microbial manipulation, and forage legumes. Figure 6 demonstrates the benefits which could be obtained from each of these activities if associated emissions abatement are eligible to earn Australian carbon credit units.

The adoption and productivity assumptions stated previously were applied to the calculation of these values. The benefits from emissions reduction payments assume a carbon price of AUD16.14/t CO2-e, the average price for each Australian carbon credit in the March 2020 Emissions Reduction Fund auction (Clean Energy Regulator, 2020) and also assumes that emission reductions for each of these activities will be eligible to receive payments. The following assumptions were used to calculate these net present values: discount rate of 7%, where this value is used to account for the time value of money, *Asparagopsis*: cost of US$7.50/kg, rate of 0.2% of feed intake; rumen microbial manipulation: US$7.50/kg, 0.2% feed to supplement intake, requires supplement for only 60 d per year; *Desmanthus*: establishment cost of US$400/ha, destock of 6 mo while establishing; *Leucaena*: establishment cost of US$400/ha, destock for 6 mo while establishing; grape marc: cost of US$84.50/t; nitrate: cost of US$2.20 per feed lick sufficient for 10 head cattle and 40 head sheep; genetics: no additional cost as assumed to be adopted as an additional desirable trait; shrubs: annualized establishment and maintenance cost of US$46.11/ha; *Biserrula*: establishment cost of US$400/ha and destock for 6 mo while establishing.

## Carbon Neutral Red Meat Systems—Practices and Feasibility

The Australian red meat industry has set a target to become carbon neutral by 2030 (The Australian Beef Sustainability Framework, 2019). Greenhouse gas emissions from the red meat sector in 2005 were 124.1 Mt CO2e and by 2015, emissions from the red meat sector had declined by 68.6 Mt CO2e. This was driven primarily by a decrease in CO_2_ emissions from reduced deforestation. In 2015, land clearing represented 30.1 Mt CO2e, whereas enteric methane emissions remained almost unchanged (Mayberry et al., 2019).

The main pathways for the industry to become carbon neutral will include reduction of methane emissions from cattle and sheep, management of wild fire savannah burning with controlled burning, widespread use of anti-methanogenic legumes and forages, decreased forest clearing, native vegetation regrowth, and new tree plantations. All of these property level activities are capable of incentivization through government backed carbon markets (ERF, 2016). To date, approximately 70% of all Australian government contracts to mitigate GHG are sited on red meat properties. This confluence of targeted research reinforced by appropriate carbon market mechanisms demonstrates the impact these practices have had and could have on farm practice. The evolving opportunities identified in this paper also indicate that red meat production will become an essential partner in the reduction of global greenhouse gas emissions.

## About the Authors

Tom Davison obtained his PhD from the University of Queensland on milk production from tropical grazing systems and led national research into heat stress management for dairy. He then held research management and investment positions with Dairy Australia and Meat and Livestock Australia. While with MLA he led the National Livestock Methane Program from 2010 to 2016 that conducted research into productivity and methane mitigation in ruminants. His membership of the Emissions Assurance Reduction Committee and understanding of carbon markets gave rise to leadership of a study into potential pathways for the Australian red meat industry to become Carbon Neutral.

John Black obtained his PhD at the University of Melbourne, Australia, quantifying the amino acid and energy requirements of growing lambs. He spent 20 yr at the Australian Commonwealth Scientific and Industrial Research Organization and the last 23 yr as Director of a Research Management Organization. His research has spanned a wide range of animal species including sheep, dairy cows, beef cattle, pigs, poultry, and honeybees. He regards himself as a reductionist scientist endeavoring to understand the mechanisms underlying biological systems, but then integrates this knowledge back into whole animal and enterprise systems using computer simulation models for practical application.

Jonathan Moss is a Lecturer in Quantitative Economics at the University of New England, Australia. Jonathan’s research interests lie in the application of bioeconomic modeling techniques, the combination of biological and economic models, to provide practical solutions to contemporary sustainability issues in the agricultural and natural resource sectors. Over the past decade, his research has had a strong focus on the impact of climate change to the agricultural sector and the design of optimal adaptation strategies to increase landholders’ resilience in the face of increasing uncertainty.
